# (*E*)-4-Chloro-*N*′-(4-hydroxy­benzyl­idene)­benzohydrazide

**DOI:** 10.1107/S1600536808027013

**Published:** 2008-08-30

**Authors:** De-Suo Yang

**Affiliations:** a Department of Chemistry and Chemical Engineering, Baoji University of Arts and Sciences, Baoji 721007, People’s Republic of China

## Abstract

The mol­ecule of the title compound, C_14_H_11_ClN_2_O_2_, displays a *trans* configuration with respect to the C=N double bond. The dihedral angle between the two benzene rings is 12.8 (3)°. In the crystal structure, mol­ecules are linked through inter­molecular O—H⋯O and N—H⋯O hydrogen bonds and C—H⋯π inter­actions, forming a three-dimensional network.

## Related literature

For related structures, see: Yang (2007 ▶, 2008*a* ▶,*b* ▶). For bond-length data, see: Allen *et al.* (1987 ▶). For related literature, see: Bernardo *et al.* (1996 ▶); Musie *et al.* (2001 ▶); Paul *et al.* (2002 ▶).
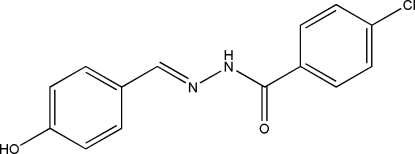


## Experimental

### 

#### Crystal data

C_14_H_11_ClN_2_O_2_
*M* *_r_* = 274.70
Orthorhombic, 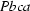

*a* = 26.251 (3) Å
*b* = 12.376 (3) Å
*c* = 7.786 (2) Å
*V* = 2529.5 (9) Å^3^
*Z* = 8
Mo *K*α radiation
μ = 0.30 mm^−1^
*T* = 298 (2) K
0.13 × 0.12 × 0.10 mm

#### Data collection

Bruker SMART CCD area-detector diffractometer
Absorption correction: multi-scan (*SADABS*; Sheldrick, 1996 ▶) *T*_min_ = 0.963, *T*_max_ = 0.971
11323 measured reflections
2164 independent reflections
1462 reflections with *I* > 2σ(*I*)
*R*_int_ = 0.078

#### Refinement

*R*[*F* ^2^ > 2σ(*F* ^2^)] = 0.047
*wR*(*F* ^2^) = 0.116
*S* = 1.02
2164 reflections
176 parameters
1 restraint
H atoms treated by a mixture of independent and constrained refinement
Δρ_max_ = 0.27 e Å^−3^
Δρ_min_ = −0.35 e Å^−3^

### 

Data collection: *SMART* (Bruker, 2002 ▶); cell refinement: *SAINT* (Bruker, 2002 ▶); data reduction: *SAINT*; program(s) used to solve structure: *SHELXS97* (Sheldrick, 2008 ▶); program(s) used to refine structure: *SHELXL97* (Sheldrick, 2008 ▶); molecular graphics: *SHELXTL* (Sheldrick, 2008 ▶); software used to prepare material for publication: *SHELXL97*.

## Supplementary Material

Crystal structure: contains datablocks global, I. DOI: 10.1107/S1600536808027013/ci2657sup1.cif

Structure factors: contains datablocks I. DOI: 10.1107/S1600536808027013/ci2657Isup2.hkl

Additional supplementary materials:  crystallographic information; 3D view; checkCIF report

## Figures and Tables

**Table 1:** Hydrogen-bond geometry (Å, °)

*D*—H⋯*A*	*D*—H	H⋯*A*	*D*⋯*A*	*D*—H⋯*A*
N2—H2*A*⋯O2^i^	0.90 (1)	2.078 (11)	2.970 (3)	171 (3)
O1—H1⋯O2^ii^	0.82	1.91	2.725 (3)	170
C6—H6⋯*Cg*1^iii^	0.93	2.88	3.726 (3)	152

Symmetry codes: (i) 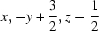; (ii) 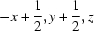; (iii) 
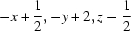
. *Cg*1 is the C1–C6 ring centroid.

**Fig. 1.**
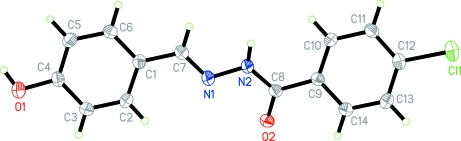
The molecular structure of the title compound, showing the atom-numbering scheme. Displacement ellipsoids are drawn at the 30% probability level.

**Fig. 2.**
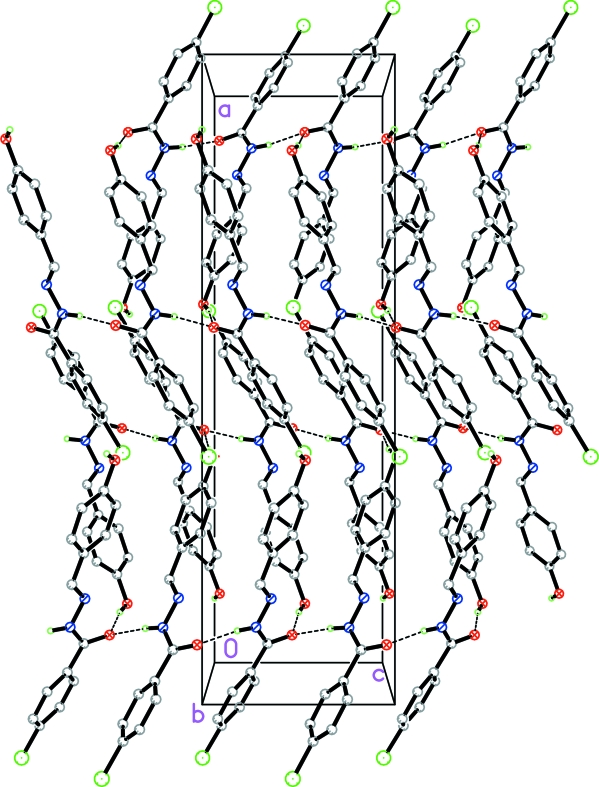
Molecular packing as viewed along the b axis. H atoms not involved hydrogen bonding (dashed lines) have been omitted for clarity.

## supplementary crystallographic information

### Comment

Schiff base compounds have been of great interest for a long time. These compounds play an important role in the development of coordination chemistry (Musie *et al.*, 2001; Bernardo *et al.*, 1996; Paul *et al.*, 2002). Recently, we have reported a few Schiff base compounds (Yang, 2007, 2008*a*,b). As a further investigation of this work, the crystal structure of the title compound is reported here.

The molecule of the title compound displays a *trans* configuration with respect to the C═N double bond (Fig. 1). The dihedral angle between the two benzene rings is 12.8 (3)°. All the bonds are within normal ranges (Allen *et al.*, 1987). The C7?N1 bond length of 1.268 (3) Å conforms to the value for a double bond. The bond length of 1.339 (3) Å between atoms C8 and N2 is intermediate between a C—N single bond and a C?N double bond, because of conjugation effects in the molecule.

In the crystal structure, molecules are linked through intermolecular O—H···O and N—H···O hydrogen bonds, and C—H···π interactions (Table 1), forming a three-dimensional network (Fig. 2).

### Experimental

4-Hydroxybenzaldehyde (0.1 mmol, 12.2 mg) and 4-chlorobenzohydrazide (0.1 mmol, 17.0 mg) were dissolved in MeOH (10 ml). The mixture was stirred at room temperature to give a clear colourless solution. Crystals of the title compound were formed by gradual evaporation of the solvent over a period of 5 days at room temperature.

### Refinement

Atom H2A was located in a difference Fourier map and refined isotropically, with N—H distance restrained to 0.90 (1) Å and with a *U*_iso_ of 0.08 Å^2^. Other H atoms were placed in idealized positions and constrained to ride on their parent atoms, with O—H distance of 0.82 Å, C—H distances of 0.93 Å, and with *U*_iso_(H) = 1.2*U*_eq_(C) and 1.5*U*_eq_(O).

### Crystal data

**Table:**

C_14_H_11_ClN_2_O_2_	*F*_000_ = 1136
*M**_r_* = 274.70	*D*_x_ = 1.443 Mg m^−^^3^
Orthorhombic, *P**b**c**a*	Mo *K*α radiation λ = 0.71073 Å
Hall symbol: -P 2ac 2ab	Cell parameters from 1916 reflections
*a* = 26.251 (3) Å	θ = 2.3–24.5º
*b* = 12.376 (3) Å	µ = 0.30 mm^−^^1^
*c* = 7.786 (2) Å	*T* = 298 (2) K
*V* = 2529.5 (9) Å^3^	Block, colourless
*Z* = 8	0.13 × 0.12 × 0.10 mm

### Data collection

**Table:**

Bruker SMART CCD area-detector diffractometer	2164 independent reflections
Radiation source: fine-focus sealed tube	1462 reflections with *I* > 2σ(*I*)
Monochromator: graphite	*R*_int_ = 0.078
*T* = 298(2) K	θ_max_ = 24.9º
ω scans	θ_min_ = 1.6º
Absorption correction: multi-scan(SADABS; Sheldrick, 1996)	*h* = −30→31
*T*_min_ = 0.963, *T*_max_ = 0.971	*k* = −14→11
11323 measured reflections	*l* = −8→9

### Refinement

**Table:**

Refinement on *F*^2^	Secondary atom site location: difference Fourier map
Least-squares matrix: full	Hydrogen site location: inferred from neighbouring sites
*R*[*F*^2^ > 2σ(*F*^2^)] = 0.047	H atoms treated by a mixture of independent and constrained refinement
*wR*(*F*^2^) = 0.116	*w* = 1/[σ^2^(*F*_o_^2^) + (0.0433*P*)^2^ + 1.3235*P*] where *P* = (*F*_o_^2^ + 2*F*_c_^2^)/3
*S* = 1.02	(Δ/σ)_max_ = 0.001
2164 reflections	Δρ_max_ = 0.27 e Å^−^^3^
176 parameters	Δρ_min_ = −0.35 e Å^−^^3^
1 restraint	Extinction correction: none
Primary atom site location: structure-invariant direct methods	

### Special details

**Table:**

Geometry. All e.s.d.'s (except the e.s.d. in the dihedral angle between two l.s. planes) are estimated using the full covariance matrix. The cell e.s.d.'s are taken into account individually in the estimation of e.s.d.'s in distances, angles and torsion angles; correlations between e.s.d.'s in cell parameters are only used when they are defined by crystal symmetry. An approximate (isotropic) treatment of cell e.s.d.'s is used for estimating e.s.d.'s involving l.s. planes.
Refinement. Refinement of *F*^2^ against ALL reflections. The weighted *R*-factor *wR* and goodness of fit *S* are based on *F*^2^, conventional *R*-factors *R* are based on *F*, with *F* set to zero for negative *F*^2^. The threshold expression of *F*^2^ > σ(*F*^2^) is used only for calculating *R*-factors(gt) *etc*. and is not relevant to the choice of reflections for refinement. *R*-factors based on *F*^2^ are statistically about twice as large as those based on *F*, and *R*- factors based on ALL data will be even larger.

### Fractional atomic coordinates and isotropic or equivalent isotropic displacement parameters (Å^2^)

**Table:**

	*x*	*y*	*z*	*U*_iso_*/*U*_eq_	
Cl1	−0.11993 (3)	0.56136 (8)	−0.02967 (12)	0.0721 (3)	
N1	0.15097 (8)	0.76262 (18)	0.3530 (3)	0.0421 (6)	
N2	0.10461 (9)	0.74598 (18)	0.2709 (3)	0.0401 (6)	
O1	0.37436 (7)	0.92023 (16)	0.5358 (3)	0.0554 (6)	
H1	0.3853	0.9786	0.5022	0.083*	
O2	0.08473 (7)	0.61751 (14)	0.4644 (2)	0.0412 (5)	
C1	0.22762 (10)	0.8643 (2)	0.3633 (3)	0.0378 (7)	
C2	0.25323 (10)	0.7913 (2)	0.4685 (4)	0.0450 (7)	
H2	0.2372	0.7273	0.5004	0.054*	
C3	0.30150 (10)	0.8119 (2)	0.5258 (4)	0.0463 (8)	
H3	0.3178	0.7630	0.5979	0.056*	
C4	0.32601 (10)	0.9058 (2)	0.4761 (3)	0.0394 (7)	
C5	0.30142 (10)	0.9795 (2)	0.3730 (4)	0.0427 (7)	
H5	0.3176	1.0435	0.3418	0.051*	
C6	0.25282 (10)	0.9580 (2)	0.3163 (4)	0.0440 (7)	
H6	0.2366	1.0075	0.2449	0.053*	
C7	0.17709 (10)	0.8413 (2)	0.2970 (4)	0.0430 (7)	
H7	0.1636	0.8853	0.2116	0.052*	
C8	0.07391 (10)	0.6686 (2)	0.3321 (3)	0.0351 (6)	
C9	0.02586 (9)	0.6472 (2)	0.2385 (3)	0.0335 (6)	
C10	−0.00001 (10)	0.7240 (2)	0.1430 (4)	0.0409 (7)	
H10	0.0134	0.7933	0.1331	0.049*	
C11	−0.04545 (10)	0.6992 (2)	0.0619 (4)	0.0441 (7)	
H11	−0.0628	0.7512	−0.0013	0.053*	
C12	−0.06436 (10)	0.5965 (2)	0.0767 (3)	0.0435 (7)	
C13	−0.03996 (10)	0.5193 (2)	0.1731 (4)	0.0476 (8)	
H13	−0.0536	0.4502	0.1827	0.057*	
C14	0.00479 (10)	0.5449 (2)	0.2554 (3)	0.0425 (7)	
H14	0.0210	0.4934	0.3229	0.051*	
H2A	0.1010 (12)	0.783 (2)	0.172 (2)	0.080*	

### Atomic displacement parameters (Å^2^)

**Table:**

	*U*^11^	*U*^22^	*U*^33^	*U*^12^	*U*^13^	*U*^23^
Cl1	0.0490 (5)	0.0779 (6)	0.0895 (7)	−0.0117 (4)	−0.0259 (5)	0.0004 (5)
N1	0.0359 (13)	0.0460 (15)	0.0443 (14)	−0.0041 (11)	−0.0089 (11)	0.0015 (12)
N2	0.0355 (13)	0.0423 (14)	0.0424 (13)	−0.0048 (11)	−0.0094 (11)	0.0047 (11)
O1	0.0404 (13)	0.0531 (14)	0.0726 (15)	−0.0084 (9)	−0.0133 (11)	0.0061 (12)
O2	0.0436 (11)	0.0403 (11)	0.0398 (10)	0.0025 (9)	−0.0063 (9)	0.0053 (9)
C1	0.0356 (16)	0.0400 (16)	0.0379 (15)	−0.0022 (13)	−0.0013 (13)	−0.0006 (13)
C2	0.0419 (17)	0.0391 (16)	0.0539 (18)	−0.0091 (13)	−0.0022 (15)	0.0060 (14)
C3	0.0428 (18)	0.0409 (17)	0.0552 (19)	−0.0001 (13)	−0.0092 (15)	0.0096 (15)
C4	0.0321 (16)	0.0425 (17)	0.0437 (16)	−0.0009 (12)	−0.0019 (13)	−0.0058 (14)
C5	0.0437 (17)	0.0371 (16)	0.0472 (17)	−0.0063 (13)	0.0020 (14)	0.0023 (14)
C6	0.0423 (17)	0.0450 (18)	0.0447 (17)	−0.0012 (14)	−0.0075 (14)	0.0094 (13)
C7	0.0400 (17)	0.0448 (18)	0.0442 (17)	−0.0007 (14)	−0.0071 (13)	0.0045 (14)
C8	0.0365 (16)	0.0323 (15)	0.0365 (15)	0.0047 (13)	−0.0008 (12)	−0.0052 (13)
C9	0.0318 (15)	0.0361 (16)	0.0325 (14)	0.0037 (12)	0.0025 (12)	0.0007 (12)
C10	0.0392 (16)	0.0353 (16)	0.0480 (16)	−0.0046 (13)	−0.0025 (14)	0.0027 (13)
C11	0.0391 (16)	0.0483 (18)	0.0449 (17)	0.0014 (14)	−0.0064 (14)	0.0077 (14)
C12	0.0333 (16)	0.0520 (19)	0.0451 (17)	−0.0032 (14)	−0.0032 (13)	−0.0031 (15)
C13	0.0441 (18)	0.0426 (18)	0.0560 (19)	−0.0116 (14)	−0.0027 (15)	0.0030 (15)
C14	0.0431 (17)	0.0408 (17)	0.0438 (16)	−0.0003 (14)	−0.0046 (14)	0.0064 (14)

### Geometric parameters (Å, °)

**Table:**

Cl1—C12	1.733 (3)	C5—C6	1.376 (4)
N1—C7	1.268 (3)	C5—H5	0.93
N1—N2	1.390 (3)	C6—H6	0.93
N2—C8	1.339 (3)	C7—H7	0.93
N2—H2A	0.899 (10)	C8—C9	1.481 (3)
O1—C4	1.363 (3)	C9—C10	1.385 (3)
O1—H1	0.82	C9—C14	1.387 (4)
O2—C8	1.242 (3)	C10—C11	1.384 (4)
C1—C6	1.385 (4)	C10—H10	0.93
C1—C2	1.392 (4)	C11—C12	1.369 (4)
C1—C7	1.451 (4)	C11—H11	0.93
C2—C3	1.367 (4)	C12—C13	1.373 (4)
C2—H2	0.93	C13—C14	1.375 (4)
C3—C4	1.383 (4)	C13—H13	0.93
C3—H3	0.93	C14—H14	0.93
C4—C5	1.377 (4)		
			
C7—N1—N2	115.4 (2)	N1—C7—H7	119.3
C8—N2—N1	118.0 (2)	C1—C7—H7	119.3
C8—N2—H2A	127 (2)	O2—C8—N2	121.5 (2)
N1—N2—H2A	114 (2)	O2—C8—C9	120.8 (2)
C4—O1—H1	109.5	N2—C8—C9	117.8 (2)
C6—C1—C2	117.9 (2)	C10—C9—C14	118.8 (2)
C6—C1—C7	120.4 (2)	C10—C9—C8	124.0 (2)
C2—C1—C7	121.6 (2)	C14—C9—C8	117.2 (2)
C3—C2—C1	121.2 (3)	C11—C10—C9	121.0 (2)
C3—C2—H2	119.4	C11—C10—H10	119.5
C1—C2—H2	119.4	C9—C10—H10	119.5
C2—C3—C4	119.8 (3)	C12—C11—C10	118.7 (3)
C2—C3—H3	120.1	C12—C11—H11	120.6
C4—C3—H3	120.1	C10—C11—H11	120.6
O1—C4—C5	123.3 (2)	C11—C12—C13	121.5 (3)
O1—C4—C3	116.6 (2)	C11—C12—Cl1	119.8 (2)
C5—C4—C3	120.1 (3)	C13—C12—Cl1	118.7 (2)
C6—C5—C4	119.6 (3)	C12—C13—C14	119.5 (3)
C6—C5—H5	120.2	C12—C13—H13	120.2
C4—C5—H5	120.2	C14—C13—H13	120.2
C5—C6—C1	121.3 (3)	C13—C14—C9	120.4 (3)
C5—C6—H6	119.3	C13—C14—H14	119.8
C1—C6—H6	119.3	C9—C14—H14	119.8
N1—C7—C1	121.5 (3)		

### Hydrogen-bond geometry (Å, °)

**Table:**

*D*—H···*A*	*D*—H	H···*A*	*D*···*A*	*D*—H···*A*
N2—H2A···O2^i^	0.90 (1)	2.078 (11)	2.970 (3)	171 (3)
O1—H1···O2^ii^	0.82	1.91	2.725 (3)	170
C6—H6···Cg1^iii^	0.93	2.88	3.726 (3)	152

Symmetry codes: (i) *x*, −*y*+3/2, *z*−1/2; (ii) −*x*+1/2, *y*+1/2, *z*; (iii) −*x*+1/2, −*y*+2, *z*−1/2.
